# High-efficiency generation of blastoids with gastrulation potential through *Gata4*-induced PrE specification in mESCs

**DOI:** 10.1038/s41421-025-00849-2

**Published:** 2025-11-25

**Authors:** Fu Guo, Chao-Fan He, Yan-Hong Xu, Huan-Huan Wu, Dao-Ming Li, Shu-Yang Jiang, Xu-Sheng Ma, Cheng Huang, Qi Gu, Peng Du, Le-Qian Yu, Gui-Hai Feng, Hong-Mei Wang, Wei Li, Xin Li

**Affiliations:** 1 State Key Laboratory of Organ Regeneration and Reconstruction, Institute of Zoology, Chinese Academy of Sciences, Beijing, China; 2 University of Chinese Academy of Sciences, Beijing, China; 3 Beijing Institute for Stem Cell and Regenerative Medicine, Beijing, China; 4 School of Advanced Interdisciplinary Sciences, University of Chinese Academy of Sciences, Beijing, China; 5 MOE Key Laboratory of Cell Proliferation and Differentiation, School of Life Sciences, Peking University, Beijing, China; 6 Peking-Tsinghua Center for Life Sciences, Academy for Advanced Interdisciplinary Studies, Peking University, Beijing, China; 7 Beijing Advanced Center of RNA Biology, Peking University, Beijing, China

**Keywords:** Embryonic stem cells, Embryonic stem cells

Dear Editor,

Blastoids are regarded as promising models for further in vivo embryonic development^1–4^. Current blastoid protocols, which are based on either co-culturing embryonic stem cells/trophoblast stem cells (ESCs/TSCs)^2,3^ or utilizing totipotency-like cells^1,5,6^, rely critically on exogenous factors such as FGF4, BMP4 and CHIR99021^1–3,5,6^ to drive lineage specification and enhance blastoid formation. However, these blastoids harbor transcriptionally ambiguous cell populations and are insufficient for primitive endoderm (PrE) specification, and mostly arrest at pre-gastrulation stages^2,3,5–7^.

Here, we establish a defined system to generate blastoids from naïve ESCs, TSCs, and i*Gata4*-induced PrE-committed ESCs (i*Gata4*-ESCs), without exogenous factors, for the first time. Therefore, we term these blastoids as iX-blastoids. This system achieves efficient blastoid formation and autonomous lineage segregation independent of extrinsic signal induction. By manipulating i*Gata4*, these blastoids achieved robust PrE specification, with the proportion of the PrE lineage resembling natural blastocysts in vivo. Moreover, these iX-blastoids were cultured in vitro to the gastrulation stage and transplanted into the mouse uterus to induce decidualization. To construct blastoids independent of exogenous factors, we design to apply a transcription factor-driven (TF-driven) lineage specification to refine the PrE lineage of blastoids. Studies have shown that inducible genetic systems overexpressing *Gata4* or *Gata6* in mouse ESCs were useful for efficiently specifying PrE-like cells^8–11^. Moreover, we utilized CellPolaris^12^, a transfer learning-based tool, to predict the most influential TFs driving ESCs to differentiate into E4.5 PrE state for refining the PrE lineage for blastoid generation (Supplementary Fig. S1a). The results showed that *Gata4* and *Gata6* were the only PrE-specific factors predicted for refining the PrE lineage for blastoid generation, and *Gata4* was the top ranked candidate gene (Supplementary Fig. S1b). Therefore, we generated an i*Gata4*-ESC line with a Tet-On system. After 3 days of doxycycline (Dox) treatment, i*Gata4*-ESCs exhibited extraembryonic endoderm (XEN) cell-like morphology (Supplementary Fig. S1c), downregulation of the pluripotency markers such as *Nanog* and upregulation of PrE markers such as *Gata4*, *Sox17* (Supplementary Fig. S1d). Immunofluorescence staining also confirmed the expression of PrE markers, including GATA4, FOXA2, and SOX17 (Supplementary Fig. S1e).

As established, the co-culture of ESCs with TSCs generated an ESC/TSC bilayer structure, where ESCs became enveloped by TSCs^2^. This ESC/TSC structure supported blastoid formation under certain stimulation. However, introducing XEN-committed cells disrupted this self-organization pattern and led to the formation of ETX-embryoids^8,9,11^, where both ESCs and TSCs were enveloped by a layer of XEN cells. When we co-cultured ESCs, TSCs and i*Gata4*-ESCs, and induced i*Gata4* expression at the first day as previously reported^8,9,11^, these three types of cells also formed ETX-embryoids (Fig. 1a). We therefore hypothesized that initiating PrE-like cell specification after ESCs/TSCs bilayer establishment would enhance PrE lineage commitment in blastoids. To test whether such a strategy would enable constructing blastoids, we aggregated ESCs, TSCs and i*Gata4*-ESCs in blastoid basal medium (BBM) and set different i*Gata4* induction durations (Fig. 1a). Surprisingly, when we initiated i*Gata4* induction at day 2 post-aggregation, these cell aggregates formed blastoids by day 3 (Fig. 1b, c; Supplementary Fig. S1h), and we found that prolonging the i*Gata4* induction duration increased blastoid formation efficiency, peaking at 80.9% with continuous induction from 0 to 3 days (Supplementary Fig. S1f, g). Thus, we designated the group IV protocol as the optimal paradigm for constructing blastoids. On day 3 of blastoid formation, the blastoids displayed comparable diameters to those of embryonic day 4.5 (E4.5) blastocysts, with a slight enlargement observed on day 4 (Supplementary Fig. S1i). Cell quantification showed that the total cell number of day 3 blastoids was similar to that of E4.5 blastocysts (Supplementary Fig. S1j). A lineage-specific cell count analysis indicated that the counts of PrE and trophectoderm (TE) lineage cells were congruent with those observed in E4.5 blastocysts (Supplementary Fig. S1k). Immunofluorescence staining further confirmed that the cellular architecture and composition of day 3 iX-blastoids closely resembled blastocysts (Fig. 1d; Supplementary Fig. S1l, m and Video S1). In summary, we establish a defined system to generate blastoids, engineering TF guided PrE specification dependent on endogenous signaling molecules without exogenous factors.

**Fig. 1:**
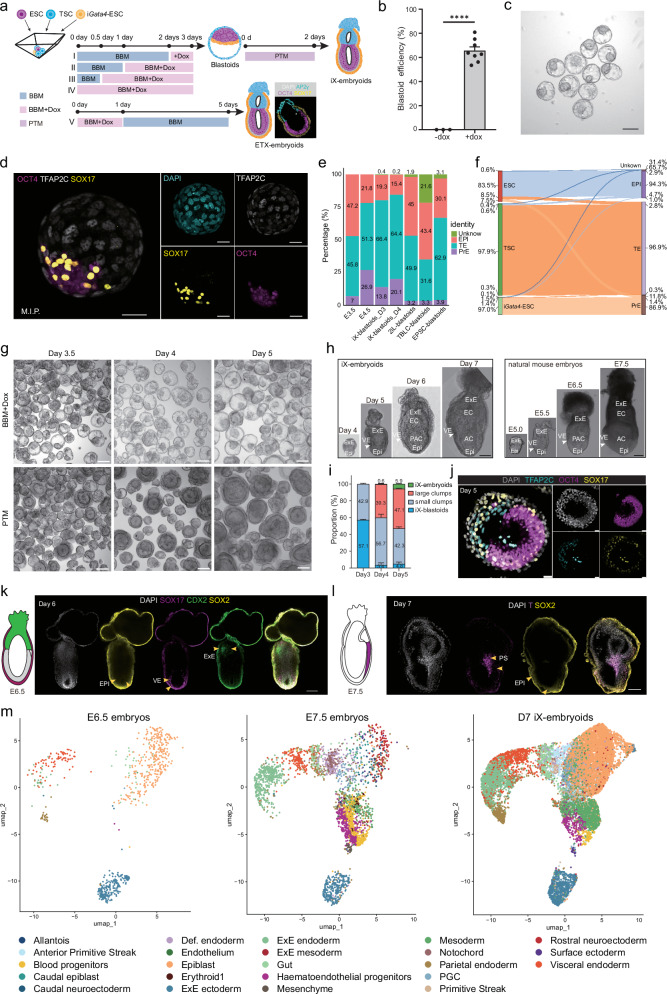
Blastoids constructed by i*Gata4*-ESCs recaptured aspects of gastrulation. **a** Temporal induction scheme for constructing iX-blastoids and ETX-embryoids using ESCs, i*Gata4*-ESCs and TSCs. The scheme also depicts the transition from iX-blastoids to iX-embryoids. BBM blastoid basal medium. PTM pluripotency transition medium. iX-embryoids, ETX-embryoids derived from iX-blastoids. **b** Quantification of iX-blastoid formation efficiency with/without doxycycline (*n* = 8, Students’ *t*-test, *p* ≤ 0.0001). Blastoid assembly efficiency was quantified as the percentage of cavitated structures relative to total cellular aggregates per AggreWell. **c** Brightfield imaging of day 4 iX-blastoids. Scale bar, 100 μm. **d** Immunofluorescence images of representative day 3 iX-blastoids. M.I.P., maximum intensity projection. Scale bar, 20 μm. **e** Quantification of cell types across different samples as shown in Supplementary Fig. S2a. **f** Sankey plot of iX-blastoids_D3, iX-blastoids_D4, and blastocysts, color coded by cell origin. Unknown, unidentified cells. Epi epiblast. TE trophectoderm. PE Primitive endoderm. TSC trophoblast stem cell. i*Gata4*-ESC Embryonic stem cell induced by *Gata4*. ESC Embryonic stem cell. natural blastocysts, E3.5 and E4.5 blastocysts. **g** Brightfield images describing the process of ETX-embryoid formation, iX-blastoids formation, and the process where iX-blastoids transited into egg cylinder-like structures under a 5-day time course. iX-blastoids underwent pluripotency transition at the indicated time points. Scale bars, 100 µm. **h** Comparative brightfield morphologies of iX-embryoids (left) vs naturally conceived embryos of corresponding stage(right). Natural mouse embryos were dissected from pregnant mice at indicated developmental stages for subsequent imaging and immunofluorescence analysis. Scale bar, 100 μm. **i** Proportional distribution of iX-blastoid-derived structures after PTM transfer (*n* = 3). Large clumps represent some disordered aggregates with volume comparable to egg cylinder-like structures. Small clumps represent disordered aggregates with volume comparable to blastoids. **j** Immunofluorescence characterization of iX-blastoids after pluripotency transition at day 5. Scale bars, 20 µm. **k, l** Immunofluorescence results showing the structures of iX-embryoids at day 6 (**k**) and day 7 (**l**). Scale bars, 100 µm. **m** UMAP visualization showing the distribution of distinct cell lineages in E6.5 embryos, E7.5 embryos, and D7 iX-embryoids.

To evaluate lineage specification within iX-blastoids, scRNA-seq was performed on day 3–4 iX-blastoids. Analyses integrating with E3.5/E4.5 blastocysts and blastoid models datasets revealed three lineages: epiblast (*Nanog*/*Pou5f1*/*Tdgf1*/*Klf4*), PrE (*Gata4*/*Gata6*/*Pdgfra*/*Sox17*), and TE (*Eomes*/*Elf5*/*Gata3*/*Gata2*) (Supplementary Fig. S2a–c). PrE proportions in iX-blastoids increased progressively from 13.8% (day 3) to 20.1% (day 4), replicating the progression observed in blastocysts from 7% (E3.5) to 26.9% (E4.5) and surpassing previous models (Fig. 1e). Hierarchical clustering analysis revealed that day 3 and day 4 iX-blastoids were closely aligned and exhibited a higher degree of transcriptional similarity to natural blastocysts than that exhibited by other models (Supplementary Fig. S2d). Notably, iX-blastoids shared a greater transcriptional similarity with E4.5 blastocysts than with E3.5 blastocysts (Supplementary Fig. S2d). Consistent with these findings, comparative lineage quantification further demonstrated that the EPI, TE, and PrE lineages in iX-blastoids exhibited stronger developmental congruence with E4.5 blastocysts than other tested models (Supplementary Fig. S2e). To elucidate the contribution of i*Gata4*-ESCs to the PrE lineage in iX-blastoids, a dual-color lineage tracing system employing mScarlet (i*Gata4*-ESCs) and GFP (TSCs) was utilized. Under this tracing strategy, GFP^+^ TSCs formed outer TE layers encapsulating inner mScarlet^+^ i*Gata4*-ESCs apical to ESC compartments (Supplementary Fig. S2f, g). Immunostaining confirmed mScarlet^+^ cells co-expressed PrE markers GATA4 and SOX17 (Supplementary Fig. S2h), validating their lineage-restricted differentiation. scRNA-seq analysis also showed that i*Gata4*-ESCs contributed 86.9% to the PrE lineage, while TSCs contributed 96.8% of TE lineages (Fig. 1f; Supplementary Fig. S2i). Concurrently, unmodified ESCs predominantly formed EPI (83.1%), with residual contributions to PrE (7.4%) and TE (8.9%) (Fig. 1f). Overall, these results validate that engineered i*Gata4*-ESCs drive PrE specification while maintaining compartmentalized lineage allocation, highlighting the autonomous lineage segregation capacity of our system.

To evaluate the developmental potential of iX-blastoids, we investigated ESC/i*Gata4*-ESC interactions under varied *Gata4* induction conditions, in ESC/i*Gata4*-ESC co-cultures, 1-day Dox treatment generated rosette architectures characteristic of post-implantation stages, while extended activation induced disorganized structures resembling pre-implantation embryos (Supplementary Fig. S3a, b). These indicate that *Gata4* dynamics govern stage-specific PrE specification and may enable iX-blastoid transition from pre-implantation to post-implantation stage. Subsequently, we transferred day 3 iX-blastoids from BBM to FGF2/Activin-A supplemented pluripotency transition medium (PTM) without Dox treatment for two days. iX-blastoids successfully transformed into egg cylinder-like structures with morphological hallmarks of E5.5 mouse embryos, with efficiency at 5.9% (117/1994, the structures derived from iX-blastoids, iX-embryoids) (Fig. 1g–i; Supplementary Fig. S3c–e). These iX-embryoids displayed a polarized EPI part (OCT4^+^) enveloped by a layer of PrE-like cells (SOX17^+^) (Fig. 1j). To assess the developmental potential of day 5 iX-embryoids, we transferred day 5 iX-embryoids to in vitro culture (IVC) medium. By day 6, these iX-embryoids formed larger egg cylinder-like structures containing SOX2⁺ epiblast and CDX2⁺ extraembryonic ectoderm (Fig. 1k). By day 7, iX-embryoids were characterized by SOX2⁺ neuroectoderm and BRA (T)⁺ primitive streak (Fig. 1l). Furthermore, we performed scRNA-seq on day7 iX-embryoids, and the analysis confirmed the presence of E7.5 stage-specific cell types and showed that their relative abundance broadly recapitulates the cellular composition of natural E7.5 embryos (Fig. 1m; Supplementary Fig. S4a, b). Additionally, expression of lineage specific markers also confirmed the specification of various cell lineages (Supplementary Fig. S4a, b). These findings demonstrate that the system recapitulates key embryonic features of the gastrulation stage. Overall, these results indicate that iX-blastoids can develop to the gastrulation and show specialization of the gastrulation stage-specific cell types.

To construct the in vivo developmental potential of iX-blastoids, we transferred iX-blastoids to the uteri of pseudo-pregnant mice. The iX-blastoids induced decidual reactions morphologically comparable to natural blastocysts, with decidualization efficiency ranging from 3.3% to 23.3% (Supplementary Fig. S5a–c). Additionally, there was an obvious maternal/fetal interface between these remnants and the uterine tissue (Supplementary Fig. S5d, e). Immunostaining revealed the expression of Sox17, Laminin, SSEA1, and Eomes in these remnants (Supplementary Fig. S5f–i). Overall, these results indicate that iX-blastoids can implant into the uterus and induce decidualization.

In summary, we have established a modular framework for assembling iX-blastoids. This approach replaces random factor-driven differentiation with precise lineage programming, enabling the development of iX-blastoids to the gastrulation stage and the induction of decidualization. Under the stimulation of PTM, the iX-blastoids successfully transformed into post-implantation stage; however, none of these iX-blastoids developed into organogenesis stage and their in vivo development is still limited. scRNA-seq analysis revealed a skewed lineage composition in D7 iX-embryoids, characterized by enrichment of epiblast and mesoderm cells, alongside a reduced abundance of specific populations such as blood progenitors and hematoendothelial progenitors (Fig. 1m). These observations may underlie the developmental limitations observed in iX-blastoids. Future efforts aimed at achieving precise control over lineage differentiation will be crucial for enhancing the fidelity of embryoid models.

## Supplementary information

Supplementary information

3D structure of iX-blastoids

## Data Availability

All datasets generated for this study have been deposited in the Genome Sequence Archive in the National Genomics Data Center, Beijing Institute of Genomics (China National Center for Bioinformation) of the Chinese Academy of Sciences (GSA: CRA021530).
