# Supplementary material for: High-efficiency generation of blastoids with gastrulation potential through Gata4-induced PrE specification in mESCs
Source: Cell Discov. 2025 Nov 25;11:94. doi: 10.1038/s41421-025-00849-2 (PMC12647567; doi:10.1038/s41421-025-00849-2)
Supplement: Supplementary file 1 — Supplementary information [file 41421_2025_849_MOESM1_ESM.pdf]

Supplementary figure S1

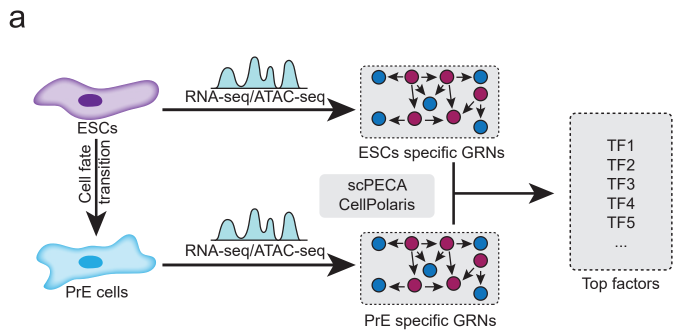

b

Top TFs	#TGs	#DEGs(%)
Gata4	7255	1171(54.0%)
Gata6	5225	884(40.1%)
Tead4	3040	596(27.5%)
Eomes	3018	577(26.6%)
Pou5f1	3006	576(26.6%)
Sox2	2895	574(25.5%)
Zfp42	2713	544(25.1%)
Klf8	2101	470(21.7%)
Klf5	1879	458(21.1%)
Klf4	1308	347(16.0%)

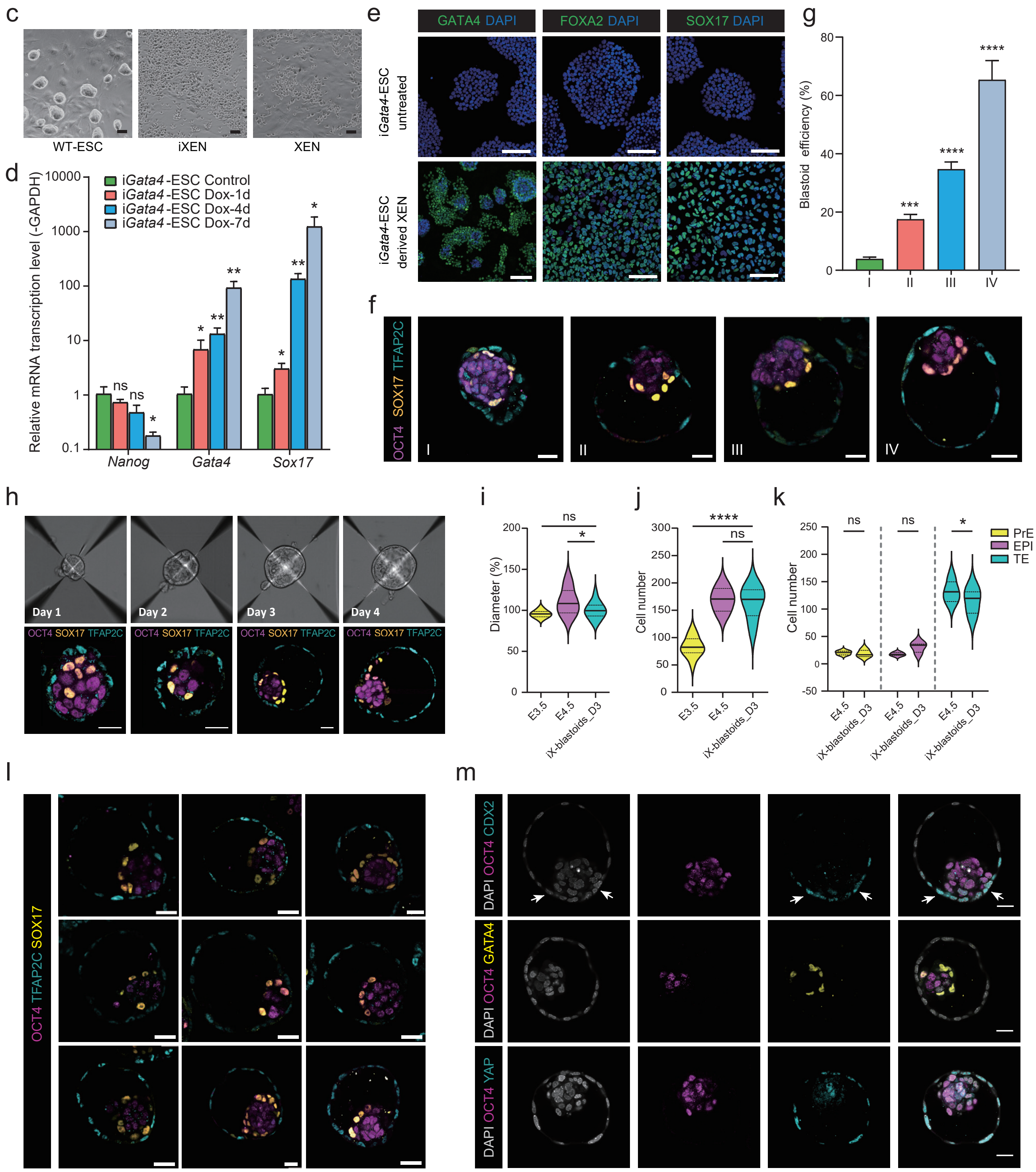

Supplementary figure S2

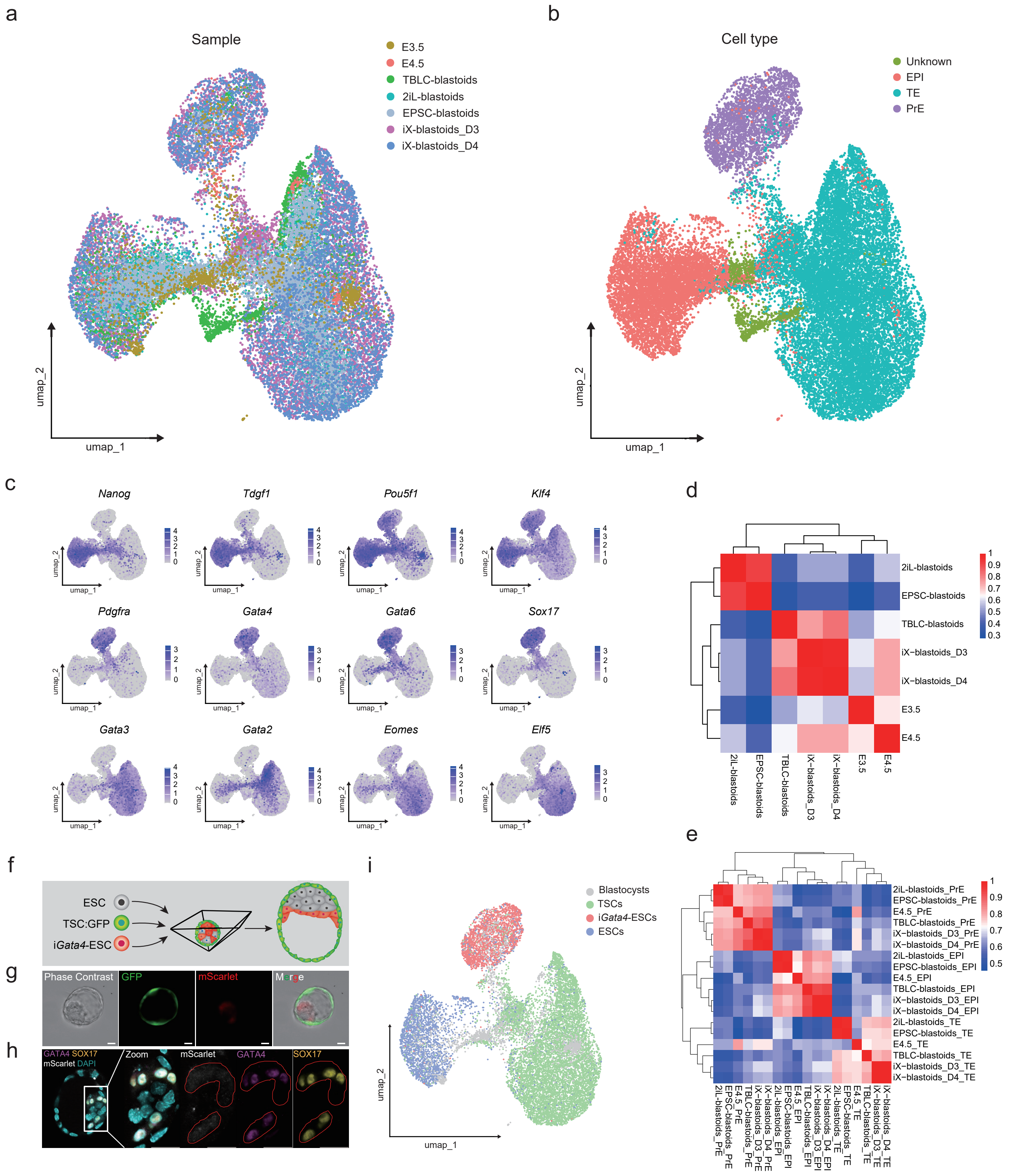

Supplementary figure S3

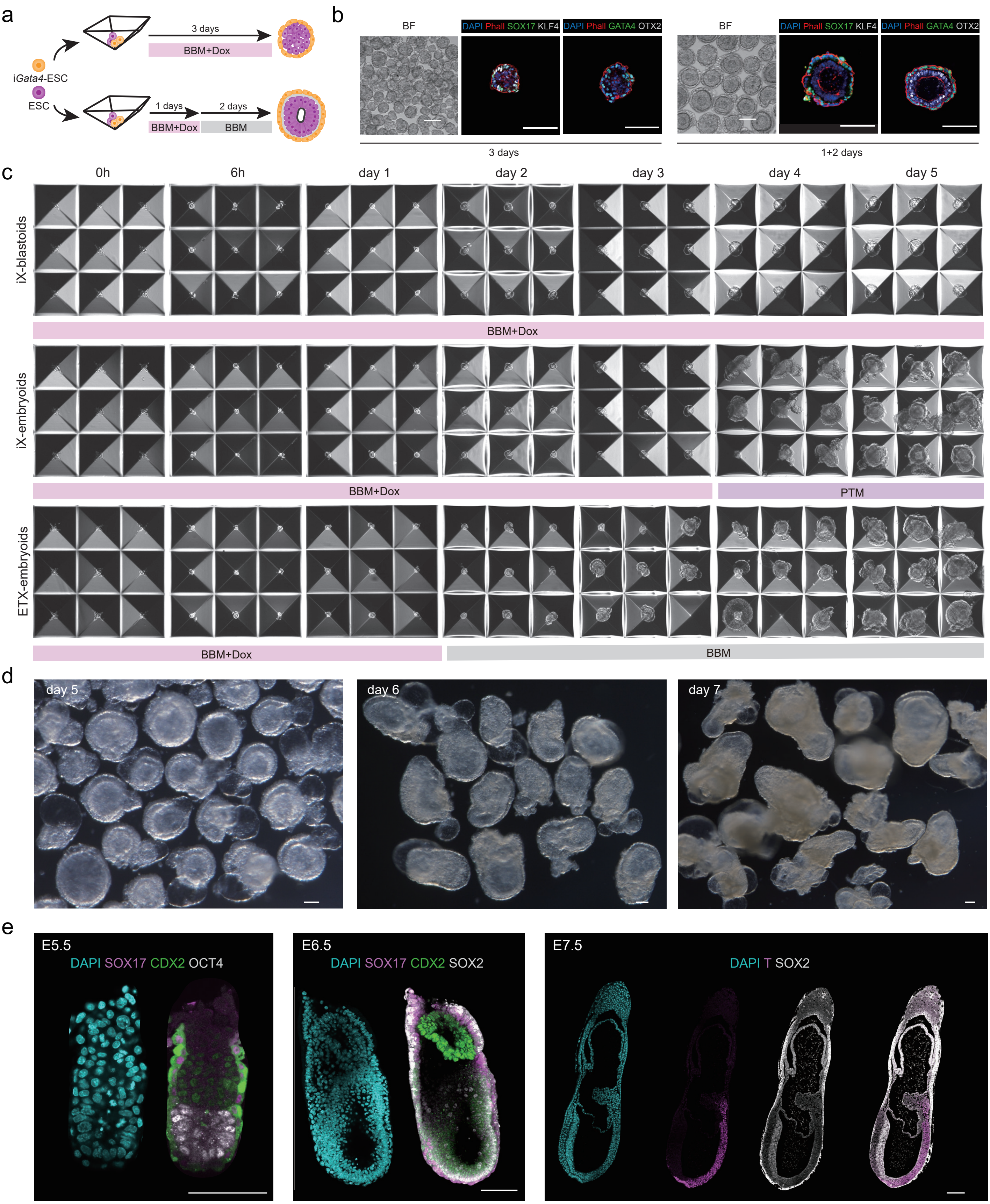

Supplementary figure S4

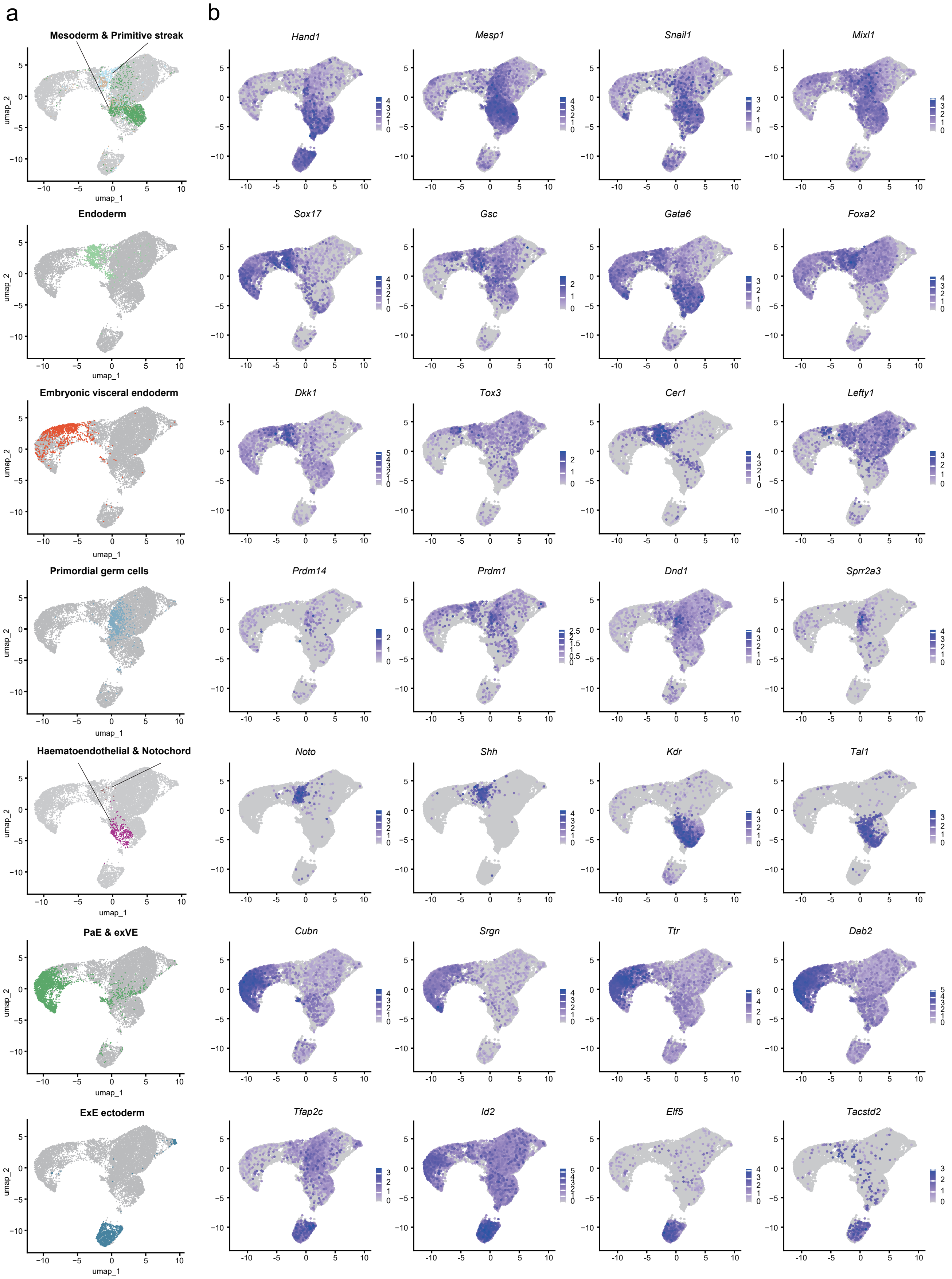

Supplementary figure S5

a

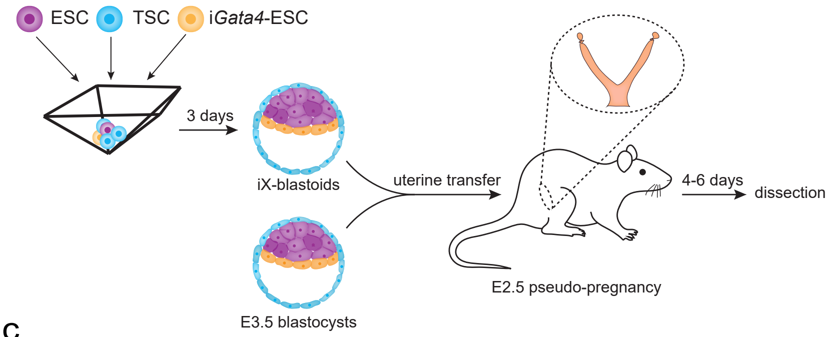

b

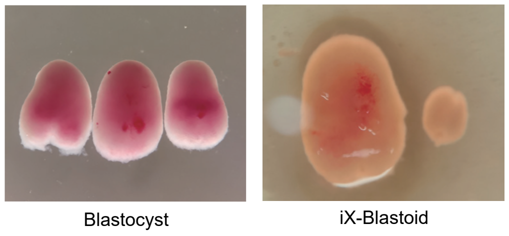

c

Sample	Days after transferred	Number of blastocysts or blastoids	Nmuber of decidua	Decidua rate (%)
E3.5 blastocyst	4	10	5	50.0
E3.5 blastocyst	5	13	10	76.9
iX-blastoid_D3	4	30	5	16.6
iX-blastoid_D3	4	30	3	10.0
iX-blastoid_D3	4	30	2	6.7
iX-blastoid_D3	4	30	1	3.3
iX-blastoid_D3	4	30	7	23.3
iX-blastoid_D3	4	30	2	6.7
iX-blastoid_D3	5	20	2	10.0
iX-blastoid_D3	5	20	1	5.0

d

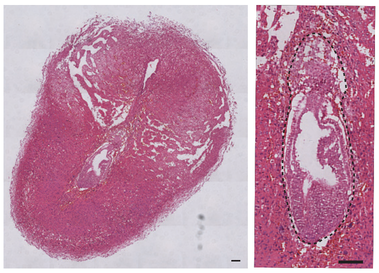

Decidua (blastocyst induced)

e

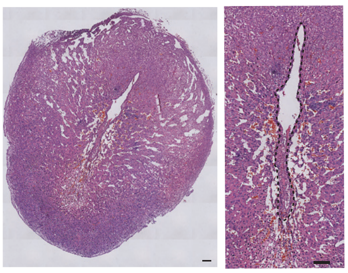

Decidua (blastoid induced)

f

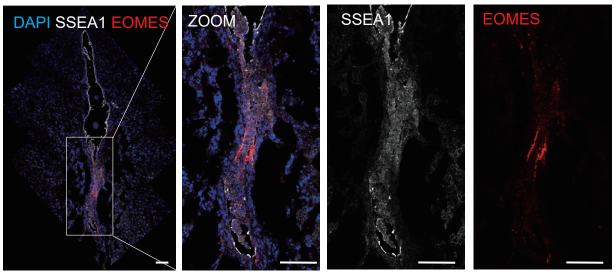

Decidua (blastoid induced)

g

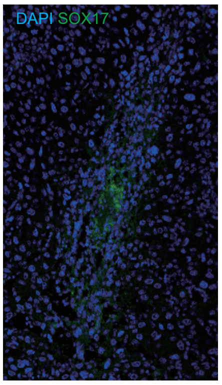

Decidua (blastoid induced)

h

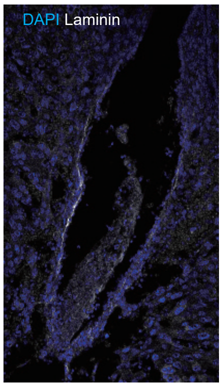

Decidua (blastoid induced)

i

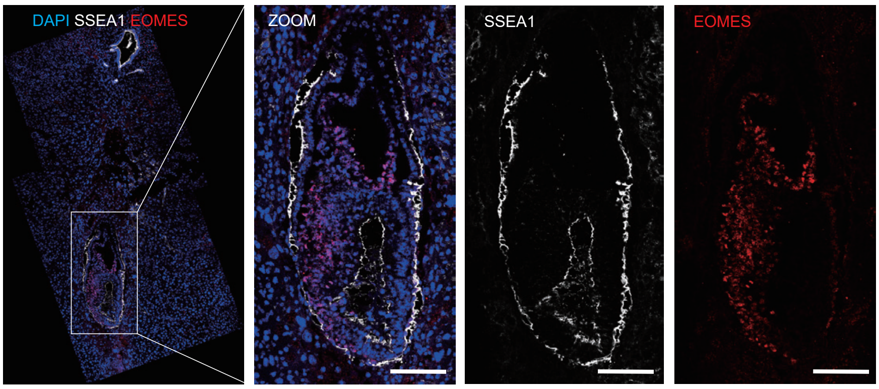

Decidua (blastocyst induced)

Supplementary Fig. S1. i*Gata4*-system boosts the formation of iX-blastoids

a CellPolaris-based prediction workflow for identifying core TFs governing PrE lineage specification in iX-blastoids. **b** Top 10 TF regulatory networks predicted by CellPolaris across fate transitions, with percentages indicating differentially expressed genes (DEG) overlaps between cell states. Top TFs: CellPolaris-predicted transcription factors; #TGs: number of target genes of specific TFs in scPECA-derived regulatory network; #DEGs (%): Quantification of differentiated expressed target genes of specific TFs (ESCs vs PrE cells), including their proportion relative to total DEGs. Red color indicates the PrE-specific TFs predicted by CellPolaris. **c** Brightfield comparisons of i*Gata4*-ESCs induced to differentiate in 2D culture with WT-ESCs and XEN cells. Scale bar, 100 μm. **d** qRT-PCR quantification of lineage-specification genes in i*Gata4*-ESCs during induction (n≥3). **e** Immunofluorescence identification of i*Gata4*-ESCs and derived PrE-like cells after *Gata4* induction for 3 days. Scale bar, 100 μm. **f** Representative immunofluorescence staining of iX-blastoids under different *Gata4* induction duration, scale bar, 20 μm. **g** iX-blastoid formation efficiency under variable *Gata4* induction durations as shown in Fig. 1A. **h** Representative phase-contrast (top) and fluorescence (bottom) images showed the formation of iX-blastoids. Scale bar, 20 μm. **i-j**. Comparative metrics between natural embryos (E3.5/E4.5) and D3 iX-blastoids: (i) Total cell counts (n ≥ 8); (j) Diameter measurements (n ≥ 9). **k** Lineage quantification in E4.5 embryos vs. D3 iX-blastoids using TE (TFAP2C), EPI (OCT4), and PrE (SOX17) markers via Imaris 9.7 (n = 8). **l, m** Representative examples of immunofluorescence staining of day3 iX-blastoids, scale bar, 20 μm. The arrows in panel M indicate polar TE cells (CDX2 possitive).

Supplementary Fig. S2. i*Gata4*-ESCs refine the PrE lineage in iX-blastoids

a, b UMAP plot representing the cells analyzed, color-coded by sample identity (a) or presumptive lineage identity (b). **c** Lineage-specific gene expression profiles of each sample according to scRNA-seq data. **d, e** Gene expression correlations: (d) inter-samples; (e) lineage-specific. Red indicated a high correlation while blue indicated a low correlation. **f** Scheme of lineage tracing strategy to trace the cell contribution during the formation of iX-blastoids. **g** Bright filed and fluorescent images of a representative iX-blastoid. Scale bar, 20 μm. **h** Immunofluorescence lineage tracing was performed on iX-blastoids to map the developmental contributions of i*Gata4*-ESCs, with multi-channel fluorescence imaging resolving cell-type-specific protein signatures across embryonic compartments. Scale bar 20 μm. **i** UMAP plots of cell-origin according to fluorescence label.

Supplementary Fig. S3. iX-blastoids transit into post-implantation stage

a Schematic representation of time-dependent *Gata4* induction effects on ESC pluripotency states, demonstrating co-culture outcomes between naive ESCs and i*Gata4*-ESCs under different induction durations. **b** Brightfield and immunofluorescent staining images of resulting structures when ESCs and i*Gata4*-ESCs were co-seeded into aggrewell under different *Gata4* induction duration as shown in Supplementary Fig. S3A. Sale bar, 100 μm. **c** Phase-contrast time-course imaging sequentially documented three processes: (1) iX-blastoid formation, (2) iX-blastoids transiting to iX-embryoids and (3) ETX-embryoid formation, showing the morphological progression of cell aggregates at

specified time points. **d** Representative brightfield images of iX-embryoids cultured *in vitro* at day 5, day 6, and day 7. Scale bar, 100 μm. **e** Immunofluorescence results showed the structures of E5.5, E6.5 and E7.5 natural embryos, corresponding to day5, day6 and day7 iX-embryoids respectively. Scale bar, 100 μm.

Supplementary Fig. S4. iX-embryoids recapture aspects of gastrulating embryos
a, b UMAP plot representing the cells analyzed, which were color-coded by sample (a) and specific lineage gene expression (b).

Supplementary Fig. S5. Developmental competent *in utero* of iX-blastoids
a Schematic of the uterine transfer assay for *in vivo* developmental potential evaluation. **b** Representative images of decidual tissues harvested 4 days post-transfer, induced by natural blastocysts (left) and blastoids (right). **c** Quantification of decidual number induced by blastocysts versus blastoids. **d, e** H&E-stained decidual tissue 4 days post-transfer of E3.5 blastocysts (d) and D3 iX-blastoids (e) into pseudopregnant mice (E2.5). Scale bar, 100 μm. **f-i** Immunofluorescence analysis of decidual tissue 4 days post-transfer of D3 iX-blastoids (f-h) and natural blastocysts (i) into E2.5 pseudopregnant mice. Scale bar, 100 μm.

Supplementary Video S1. 3D structure of iX-blastoids
3D rotational view of a day 3 iX-blastoid, reconstructed from confocal z-stack images. Scale bar, 20 μm.

Supplementary materials and methods

Animal care and use

Mice (Approval number: IOZ-IACUC-2024-191) and rats (Approval number: IOZ-IACUC-2024-012) were housed in the animal care facility at the Institute of Zoology, Chinese Academy of Sciences, in accordance with institutional guidelines for the care and use of laboratory animals. ICR mice for embryo recovery were purchased from Beijing Vital River Laboratory Animal Technology Co., Ltd. (Beijing, China). All animal research and care procedures were approved by the Institutional Animal Use Committee of the Institute of Zoology at the Chinese Academy of Sciences, Beijing, China.

For cell recovery, female mice aged 6–8 weeks were naturally mated, and the morning a vaginal plug was observed this morning was designated as E0.5. Mice were euthanized via cervical dislocation on day 3, 4, 5, 6, and 7 post-coitum. Embryos were obtained by flushing the uterine or dissecting decidua, depending on the implantation stage.

For uterine transfer assay, vasectomized ICR male mice (8-12 weeks old) were co-housed with ICR females (6-8 weeks old) overnight. Females exhibiting vaginal plugs the following morning were designated as pseudopregnant recipients, with plug detection designated as E0.5. At E2.5, blastocysts (15-20 per uterine horn) or blastoids (20-30 per horn) were surgically transferred into contralateral uterine horns via oviduct infusion. Decidual tissues were collected at E6.5 and E7.5.

Serum

The experiments for rat serum (RS) obtaining and purification followed the guidelines of the Animal Care and Use Committee of the IOZ, CAS. The human cord serum (HCS) was obtained under the approval given by the Research Ethics Committee of the Beijing Obstetrics and Gynecology Hospital. The donors of the placental samples used in this study were healthy pregnant women without any exclusion. All donors in our research project shared one ethical approval license number (research license number: 2022-KY-074-01).

Cell culture

All stem cells were cultured on a feeder layer of mitomycin-treated CF1 mouse embryonic fibroblasts (MEFs) under conditions of 20% O_2 and 5% CO_2 at 37℃. All cells used for blastoid production were maintained in 12-well Plates (3516, Corning).

Naive ESCs: ESCs (129×129 and ICR×ICR) and i*Gata4*-ESCs (129×129 and ICR×ICR) were cultured in N2B27 basal medium supplemented with 1 μM PD0325901 (04-0006-10, Stemgent), 3 μM CHIR99021 (04-0004-10, Stemgent) and 10 ng/mL Mouse recombinant LIF (ESG1107, Merck Millipore). N2B27 basal medium is composed of a 1:1 mix of DMEM/F12 (11320-033,Thermo Fisher Scientific) and Neurobasal (21103-049, Thermo Fisher Scientific) supplemented with 1% v/v B27 Supplement (17504044, Thermo Fisher Scientific), 0.5% v/v N2 Supplement (17502048, Thermo Fisher Scientific), 0.1% 2-Mercaptoethanol (21985023, Thermo Fisher Scientific), 1% penicillin-streptomycin (15140122, Thermo Fisher Scientific),1% MEM NEAA (11140050,Thermo Fisher Scientific), 1% GlutaMAX (35050-061, Thermo Fisher Scientific) and 5% KnockOut Serum Replacement. Cells were routinely passaged every two days using 0.05% Trypsin-EDTA (25300062, Thermo Fisher Scientific).

TSCs: TSCs derived from 129 and ICR mouse strains were maintained in TS conditioned medium (TS-CM). The TS-CM consisted of a mixture of 70% MEF conditioned medium (MEF-CM) and 30% TS basal medium, supplemented with 25 ng/mL FGF4 (5846-F4, R&D Systems) and 1 μg/mL heparin (H3149, Sigma).TS basal medium was RPMI-1640 Medium (11875093, Thermo Fisher Scientific) supplemented with 20% Fetal Bovine Serum (10099141, Gibco), 2-Mercaptoethanol (21985023, Thermo Fisher Scientific), 1% Sodium Pyruvate (11360070, Thermo Fisher Scientific),1% L-Glutamine (25030081, Thermo Fisher Scientific) and 1% penicillin-streptomycin (15140122, Thermo Fisher Scientific). Cells were routinely passaged every three days using 0.05% Trypsin-EDTA (25300062, Thermo Fisher Scientific).

For differentiation of PrE-like cell from i*Gata4*-ESCs, the culture medium was removed and the plate was washed by PBS to remove residual medium. Later, TS basal medium supplemented with 10 nM Dox was used to induce i*Gata4* expression.

Plasmid construction and electroporation of i*Gata4*-ESCs

Plasmid backbone Rosa26-tetOn-WPRE-mScarlet-NeoR was constructed by our lab. The targeting plasmid incorporates homology arms for Rosa26 locus integration, sequentially encoding the following genetic elements: (1) a CAG promoter-regulated reverse tetracycline-controlled transactivator (rtTA), (2) a tetracycline response element (TRE)-controlled miniCMV promoter module, and (3) a woodchuck hepatitis virus post-

transcriptional regulatory element (WPRE) flanking the transgene. Selection markers consist of a human EF1α promoter-driven neomycin resistance cassette (NeoR) co-expressed with mScarlet fluorescent protein via P2A peptide linkage. *Gata4* cDNA (Youbao Bio) was directionally cloned into the multiple cloning sites through HiFi DNA assembly (NEB), with vector linearization achieved by Cla I (R0197V, NEB) and Nhe I (R3131L, NEB) restriction digestion. Electroporation-mediated transfection was performed using the Neon Transfection System (Invitrogen) following standard protocols.

RNA Extraction and qRT-PCR

Total RNA was extracted from the centrifuged cell pellets with Trizol. (15596026, Invitrogen). Reverse transcription was conducted using qPCR RT Master Mix (FSQ-201, Toyobo). For fluorescent quantitative PCR, the LightCycler 480 System (Roche) was employed with Universal Blue qPCR SYBR Green Master Mix (11184ES60, Yeasen). All procedures were performed according to the manufacturer's instructions. Gene expression levels were normalized to *Gapdh* and analyzed via the $\Delta\Delta$CT method for quantification of relative transcript abundance.

Generation of iX-blastoids

ESC, i*Gata4*-ESC, or TSC colonies were dissociated into single cells by 0.05%Trypsin-EDTA (see above). After centrifuged, single cells were resuspended in TS basal medium and seeded onto plates coated with 0.1% gelatin, then incubated at 37°C for 30 minutes to allow irradiated MEF cells to attach to the plates. The supernatant containing the single cells was then collected and counted using a counter (A49891, Invitrogen). AggreWell 400 (34415, STEMCELL Technologies) was prepared according to the manufacturer's protocols. iX-blastoid basal medium (BM) was composed of 25% N2B27 basal medium, 25% TS basal medium, and 50% KSOM (M1435, Nanjing Aibei). Suspensions of ESCs (6000 cells per well), i*Gata4*-ESCs (6000 cells per well), and TSCs (18000 cells per well) were mixed into BM, supplemented with 2 mM ROCK inhibitor Y-27632 (HY-10071, MCE) and 10 μM Doxycycline (HY-N0565, MCE), and seeded into a single well of the AggreWell plate. The plate was centrifuged at 200 g for 2 minutes, and placed in an incubator. Y-27632 was removed from the medium after 12 hours by replacing the medium with BM supplemented only with Dox. iX-blastoids were cultured at 37℃ in an atmosphere of 20% O_2 and 5% CO_2. Samples were collected at different time points for downstream experiments by manually picking them up using a mouth pipette under a stereomicroscope, as needed for the experiment.

Pluripotency transitions of iX-blastoids

The transition process began on day 3 of iX-blastoid formation, with the medium replaced by N2B27 basal medium which was supplemented with 12 ng/ml FGF2 (HY-P7004, MCE) and 10 ng/ml Activin A (C687, Novoprotein). The transition was conducted at 37℃ under 20% O_2 and 5% CO_2 conditions for two days. These egg cylinder-like piX-blastoids were manually collected using a mouth pipette under a stereomicroscope for further culture or immunofluorescence staining.

Extended culture of iX-blastoids

At day 5, egg cylinder-like iX-embryoids were picked and transferred to 24 well low attachment well plates (Corning, 3473; 50-100 iX-embryoids per well) containing pre-equilibrated 500 μL of freshly prepared culture media and were cultured in a humidified incubator with 5% CO2 and 20% O_2 at 37°C. On day 6 and day 7, half of the pre-equilibrated culture medium was refreshed. The culture media originally included IVC and EUCM conditions and was composed of 25% CMRL 1066 (Gibco, 11530037) plus 50% rat serum and 25% human cord serum (HCS) that was prepared in-house, and supplemented with a final concentration of 1× ITS-X (Thermo Fisher, 51500056), 1× penicillin–streptomycin (Yeasen, 60162ES76), 1× GlutaMAX (Thermo Fisher, 35050061), 1× sodium pyruvate (Gibco, 11360070), 8 nM β-estradiol (Sigma-Aldrich, E8875), 200 ng/mL progesterone (Sigma-Aldrich, V900699) and 25 μM N-acetyl-l-cysteine (Sigma-Aldrich, A9165). Rat serum and HCS were heat inactivated at 56°C for half an hour and filtered through a 0.22 mm PVDF filter (Merck Millipore, GSWP02500), and then stored at -80°C for up to 6 months. Rat serum and HCS should avoid repeated freeze-thaw before experimentation.

Efficiency statistics

All statistical analyses were performed using GraphPad Prism 9.3.1. Intergroup comparisons were conducted via unpaired two-tailed Student's t-test with Welch's correction for unequal variances. Significance thresholds were defined as: *p < 0.05, **p < 0.01, ***p < 0.001. Error bars represent mean ± standard error of mean (SEM).

Immunofluorescence staining

For cell staining, the cells were first fixed with 4% paraformaldehyde at room temperature for 15 minutes, washed three times in PBS (PB180327, Pricella), and followed by permeabilization with permeabilize buffer (PBS containing 0.5% Triton X-100(GC204003, Servicebio)) for another 15 minutes. Then, after blocking at room temperature for 1 hour with blocking buffer (PBS supplemented with 5% donkey serum (SL050, Solarbio)), the samples were incubated overnight at 4°C with a primary antibody diluted 1:200 in the blocking buffer. The next day, the cells were washed three times with washing buffer (PBS containing 0.01% Tween-20 (T104863, Aladdin)). They were then incubated at room temperature for 1.5 hours with a secondary antibody diluted 1:500 in the washing buffer along with DAPI (D1306, Thermo Fisher Scientific). After another three washes of 5 minutes each with the washing buffer, the cells were mounted with an anti-fade mounting medium and imaged.

For iX-blastoids, rosette-like structures, and blastocysts staining, the samples were first fixed with 4% paraformaldehyde at room temperature for 30 min and washed three times in PBS. Then samples were permeabilized with permeabilized buffer for 2 hours and blocking with blocking buffer for another 2 hours at room temperature. The primary antibody was incubated overnight at 4°C in the blocking buffer. The secondary antibody and DAPI were co-incubated overnight at 4°C. The following day, samples were washed three times with washing buffer and then transferred into drops in confocal dishes (D35-10-0-N, Cellvis) sealed with mineral oil before imaging.

For iX-embryoids and post-implantation embryos whole-mount staining, the samples were first fixed with 4% paraformaldehyde at room temperature for 2 hours. For the permeabilization process, samples were incubated overnight at 4°C in a permeabilized buffer. After blocking for 3 hours with blocking buffer, the primary antibody was incubated overnight at 4°C. Following washes, the secondary antibody and DAPI were also incubated overnight at 4°C. Finally, the samples were washed with washing buffer and prepared for imaging in drops within oil-sealed confocal dishes.

The antibodies used were: OCT4 (ab19857, Abcam), TFAP2C (sc-12762, Santa Cruz), SOX17(AF1924, R&D Systems), GATA4(sc-25310, Santa Cruz), CDX2 (MU392A-5UC, BioGenex), Yap1(sc-101199, Santa Cruz), Foxa2 (AF2400, R&D Systems) and SOX2 (ab97959, Abcam). Image acquisition was performed using Leica STELLARIS 5 and Zeiss 880 microscopes. The images were processed using the corresponding software for Leica or Zeiss microscopes. Imaris software was used for the 3D reconstruction of blastoids and blastocysts and cell count analysis.

Histological Analysis and Immunofluorescence Staining

Decidual tissues induced by blastocysts and blastoids were collected and fixed in 4% paraformaldehyde at room temperature. Samples were paraffin-embedded and sectioned at 5 μm using a Leica RM2125 RTS manual rotary microtome. Sections were deparaffinized in xylene and rehydrated through a graded ethanol series.

Hematoxylin and eosin (H&E) staining was performed using a commercial kit (Servicebio, G1005) according to the manufacturer’s instructions. After staining, sections were dehydrated, cleared, and mounted with neutral balsam. Bright-field images were acquired using a Leica Aperio VESA8 microscope.

For immunofluorescence staining, sections were subjected to antigen retrieval in 0.01 mol/L citrate buffer (pH 6.0), followed by blocking, incubation with primary antibodies, PBS washing, incubation with fluorescently labeled secondary antibodies, and nuclear counterstaining with DAPI. After staining, sections were mounted with an anti-fade mounting medium. Fluorescence images were captured using a Zeiss LSM 880 confocal microscope.

Data preprocessing and quality control

Raw scRNA-seq data was processed using Cellranger (v7.2.0), which included deduplication, alignment to the mouse reference genome (GRCm39), and the generation of a cell-by-gene count matrix. The data was then imported into Seurat R package (v5.1.0) to create a Seurat object. Gene expressed in fewer than three cells was filtered out, and low-quality cells were excluded based on the following criteria: a minimum of 200 detected genes and less than 5% mitochondrial gene content.

Normalization, dimensionality reduction, and clustering

The data was normalized using Seurat’s NormalizeData function. Principal component analysis (PCA) was performed on the top 3000 variable genes. For dimensionality reduction, PCA was conducted using the top 2000 variable genes. To correct batch effects, the Harmony package (v1.2.0) was applied, which adjusts the PCA embeddings using the

first 30 principal components. After batch correction, clustering and further dimensionality reduction were conducted using the first 30 Harmony-corrected principal components, followed by uniform manifold approximation and projection (UMAP).

Batch correction

We performed batch correction using Harmony (Korsunsky et al., 2019) to integrate single-cell RNA-seq data from multiple donors, which employed a soft clustering approach to iteratively align datasets in reduced dimensional space while preserving biological variation. The input data was log-normalized and scaled prior to dimensionality reduction. Principal component analysis (PCA) was first computed using the top 2000 highly variable genes (n = 30 principal components). Harmony integration was performed using default parameters (theta = 2, lambda = 1) with 20 maximum iterations, which achieved convergence as confirmed by the stability of integration scores across iterations. No covariates were included in the integration (vars.use = NULL) to focus correction specifically on technical batch effects. The resulting Harmony-corrected embeddings were used for all downstream analyses including clustering and visualization.

PECA2 GRN construction and PGM-GRN modeling

We have meticulously curated a cell type-specific PECA2 network by integrating single-cell RNA sequencing (scRNA-seq) and single-cell assay for transposase-accessible chromatin using sequencing (scATAC-seq) datasets. Our dataset comprises ES cells from our scRNA-seq and scATAC-seq[1], E4.5 blastocysts cells from scRNA-seq[2], and XEN cells from scATAC-seq[1]. For each cell type, we aggregated scRNA-seq data to generate pseudo-bulk RNA-seq data, calculating FPKM values. Similarly, we normalized the aggregated scATAC-seq data to reflect chromatin accessibility, termed as cell type openness data. This comprehensive approach yielded paired pseudo-bulk datasets for RNA-seq and ATAC-seq, which were instrumental in constructing the PECA2 network. We then model PECA2 genes and regulatory relationships as part of the input to construct the PGM model in CellPolaris.

Calculate the number of differential genes to identify the important TFs

To find important TFs, we assessed gene expression alterations post-knockout. The initial FPKM expression profile of the cluster was denoted as vector e1, the deltaX expression post-knockout as vector e2, and another cluster expression FPKM vector is recorded as ec. We then determined the intersection of TFs active in both ES and E4.5 stages and calculated the proportion of differential genes regulated by these TF in the total number of differential genes (with |logFC| >= 2). This rigorous computational strategy unveils the regulatory landscape governing cell fate decisions and highlights the significance of specific TFs in these processes.

Doublet detection and cell type annotation

After initial clustering, potential doublets were identified by examining cells that expressed marker genes of multiple cell types. DoubletFinder (v2.0.4) was used to estimate the expected doublet rate, and cells were classified based on their doublet probabilities. The

"Singlet" cells were retained for downstream analysis. Following doublet removal, the dataset underwent re-clustering and dimensionality reduction using the same methods as described previously. Cell clusters were annotated by referencing known cell-type marker genes.

Automated reference-based cell type annotation

The cell type annotation for the scRNA-seq data presented in Fig. 1M and Supplementary Fig. S4 was performed using SingleR (v2.4.1)[3], an automated reference-based annotation algorithm. For annotation, we selected the dataset from published data[4] as our reference, which contains comprehensive single-cell transcriptomic data of natural mouse embryos at different developmental stages.

The annotation procedure was implemented as follows: The reference expression matrix was input via the 'ref' parameter of the SingleR function, while the corresponding cell type labels of the reference dataset were supplied to the 'labels' parameter. Our query scRNA-seq dataset was assigned to the 'test' parameter. The algorithm computes the Spearman correlation between each cell in the test set and all cells in the reference dataset based on gene expression profiles, subsequently assigning the cell type label of the best-matching reference cell to each test cell. The annotation was performed using the default mode of the algorithm.

Data visualization

Data visualization was performed using Seurat and ggplot2 packages in R. UMAP plots were generated to illustrate the distribution of cells across different clusters. To ensure comparability across samples, cell numbers for each group were down-sampled to the same level.

Statistical analysis and reproducibility

No specific statistical methods were used for sample size estimation. The number of samples was determined by sample availability and in reference to similar published studies. Data analysis, including differential gene expression analysis, was performed using Seurat. All statistical tests were two-tailed, and p-values less than 0.05 were considered statistically significant.

1. Garg, V., Yang, Y., Nowotschin, S., Setty, M., Salataj, E., Kuo, Y.Y., Murphy, D., Sharma, R., Jang, A., Polyzos, A., et al. (2025). Single-cell analysis of bidirectional reprogramming between early embryonic states identify mechanisms of differential lineage plasticities in mice. Dev Cell *60*, 901-917.e912. 10.1016/j.devcel.2024.11.022.
2. Nowotschin, S., Setty, M., Kuo, Y.Y., Liu, V., Garg, V., Sharma, R., Simon, C.S., Saiz, N., Gardner, R., Boutet, S.C., et al. (2019). The emergent landscape of the mouse gut endoderm at single-cell resolution. Nature *569*, 361-367. 10.1038/s41586-019-1127-1.
3. Aran, D., Looney, A.P., Liu, L., Wu, E., Fong, V., Hsu, A., Chak, S., Naikawadi, R.P., Wolters, P.J., Abate, A.R., et al. (2019). Reference-based analysis of lung single-

cell sequencing reveals a transitional profibrotic macrophage. Nat Immunol *20*, 163-172. 10.1038/s41590-018-0276-y.
4. Pijuan-Sala, B., Griffiths, J.A., Guibentif, C., Hiscock, T.W., Jawaid, W., Calero-Nieto, F.J., Mulas, C., Ibarra-Soria, X., Tyser, R.C.V., Ho, D.L.L., et al. (2019). A single-cell molecular map of mouse gastrulation and early organogenesis. Nature *566*, 490-495. 10.1038/s41586-019-0933-9.
